# Pectin Extraction from Residues of the Cocoa Fruit (*Theobroma cacao* L.) by Different Organic Acids: A Comparative Study

**DOI:** 10.3390/foods12030590

**Published:** 2023-01-30

**Authors:** Jenny Paola Jarrín-Chacón, Jimmy Núñez-Pérez, Rosario del Carmen Espín-Valladares, Luis Armando Manosalvas-Quiroz, Hortensia María Rodríguez-Cabrera, José Manuel Pais-Chanfrau

**Affiliations:** 1 Carrera de Agroindustria, FICAYA, Universidad Técnica del Norte (UTN), Ave. 17 de Julio 5-21 & José María de Córdova, Ibarra 100115, Ecuador; 2 School of Chemical Sciences and Engineering, Yachay Tech University, Hacienda San José s/n y Proyecto Yachay, Urcuqui 100119, Ecuador

**Keywords:** pectin, cocoa pod husk valorisation, citric acid, malic acid, fumaric acid, response surface methodology, central composite design

## Abstract

Ecuador is the world’s fifth largest cocoa producer, generating hundreds of tons of residues from this fruit annually. This research generates value from the residual (cocoa pod husk) by using it as raw material to obtain pectin, which is widely used in the food and pharmaceutical industries. Extraction of three different organic acids with GRAS status (safe for use), the citric, malic and fumaric acids, was studied. In addition, two other factors, temperature (70–90 °C) and extraction time (60–90 min), were explored in a central composite design of experiments. We determined the conditions of the experiments where the best yields were garnered for citric acid, malic acid and fumaric acid, along with a ~86 min extraction time. The temperature did not show a significant influence on the yield. The pectin obtained under optimal conditions was characterised, showing the similarity with commercial pectin. However, the equivalent weight and esterification degree of the pectin obtained with fumaric acid led us to classify it as having a high equivalent weight and a low degree of esterification. In these regards, it differed significantly from the other two acids, perhaps due to the limited solubility of fumaric acid.

## 1. Introduction

Ecuador is the fifth largest producer of cocoa in the world, with more than 327,000 tons per year of cocoa beans [1], and one of the leading exporters of fine-flavour cocoa, reaching 65% of the market in 2017 [2] and 54% in 2020 [3].

The chocolate paste is obtained from the roasted seeds of the cocoa fruit, which only represent about 10% of the weight of the whole fruit [4]. Thus, in Ecuador, more than 295,100 tons of residues from cocoa production are generated per year.

Cocoa residues are made up of the cocoa pod husk (CPH), the mucilage that covers the seeds and the cover that comes off when the latter is roasted [5]. However, the majority component of the residues is formed by the CPH, reaching between 90–93% wt. of the total cocoa residue [5].

CPH is formed mainly by cellulose (35.0%), lignin (14.6%), hemicellulose (11.0%), pectin (6.1%) and proteins (5.9%), in addition to mineral salts, ash and water [6,7].

Pectin, meanwhile, is the natural polysaccharide that forms part of cell walls and tissues in higher plants. It is essential in plant physiology, defence and typical development [8]. In addition, pectin gives plant tissues their mechanical resistance and flexibility [9].

Pectin has numerous uses in the food, cosmetic and pharmaceutical industries [10,11]. For example, it has been used as a gelling and thickening agent [12], and it has also been part of the formulation of food [13], cosmetics [11] and pharmaceutical applications [14,15,16,17,18,19].

Interestingly, pectin can be obtained from agricultural and food waste [20,21,22,23,24], which considerably lowers its production costs and reduces the environmental impact these wastes can exert on ecosystems [25,26,27,28].

CPH can be an attractive raw material for obtaining cellulose [29,30], antioxidant compounds [31] and pectin [9,21,32]. However, the pectin extraction yields from CPH depend on several factors [33]. For example, the chosen solvent, the solvent/CPH ratio used, the extraction temperature and the duration of the process, among others, influence the extraction process yields [34]. 

CPH-pectin can be extracted using acid solvents, and the extraction process can be assisted using microwaves [35,36] and enzymes [37]. Various acids have been used such as hydrochloric [33] and nitric [38] acids, and some organic acids such ascorbic [39], oxalic [36], acetic and citric [40] acids. Among all, the latter is the most common choice.

The response surface methodology (RSM) is an experimental design and analysis tool aimed at finding the conditions for which one or several responses of an experiment are optimised [41,42]. It has been used successfully in the optimisation of numerous agro-industrial processes [43], and the extraction of pectin has not been an exception [36,44,45,46,47].

This work aims to find, through the RSM, the best temperature and contact time conditions that maximise the yield of pectin extraction from dehydrated CPH using three organic acids. One already used before is citric acid, and two other organic acids are considered with GRAS status: malic and fumaric acids.

## 2. Materials and Methods

### 2.1. Raw Materials

The cocoa fruits in this work belong to the CCN-51 variety and come from the Lita Parish, Ibarra Canton, Imbabura Province, Ecuador.

### 2.2. Chemicals

Food-grade reagents citric acid monohydrate (C_6_H_8_O_7_·H_2_O, CAS 5949-29-1, food additive code: E330), L-malic acid (C_4_H_6_O_5_, CAS 97-67-6, E296) and fumaric acid (C_4_H_4_O_4_, CAS 110-17-8, E297) were supplied by Sucroal S.A. (https://sucroal.com.co (accessed on 9 January 2023), Recta Cali, Valle del Cauca, Colombia).

### 2.3. Preparation of Dehydrated Cocoa Pod Husk

All the experiments were carried out in Cayambe, Pichincha Province, Ecuador. The city is located at the coordinates 0°02′38″ N 78°09′22″ W and 2830 m above sea level.

The cocoa residues were washed with abundant tap water to remove any debris or traces of dirt on their surface, and then after drying they were weighed. 

When cutting the shell, it darkened rapidly, perhaps evidence of oxidation or maybe due to the action of hydrolytic enzymes. The enzymatic inactivation process was carried out, adding water to cover the wet residuals, and then they were heated up to 75 °C for 8 min. Subsequently, they were drained and cut into small pieces 3 mm wide to facilitate drying at 50 °C for about six hours until the residues turned amber.

**Figure 1:**
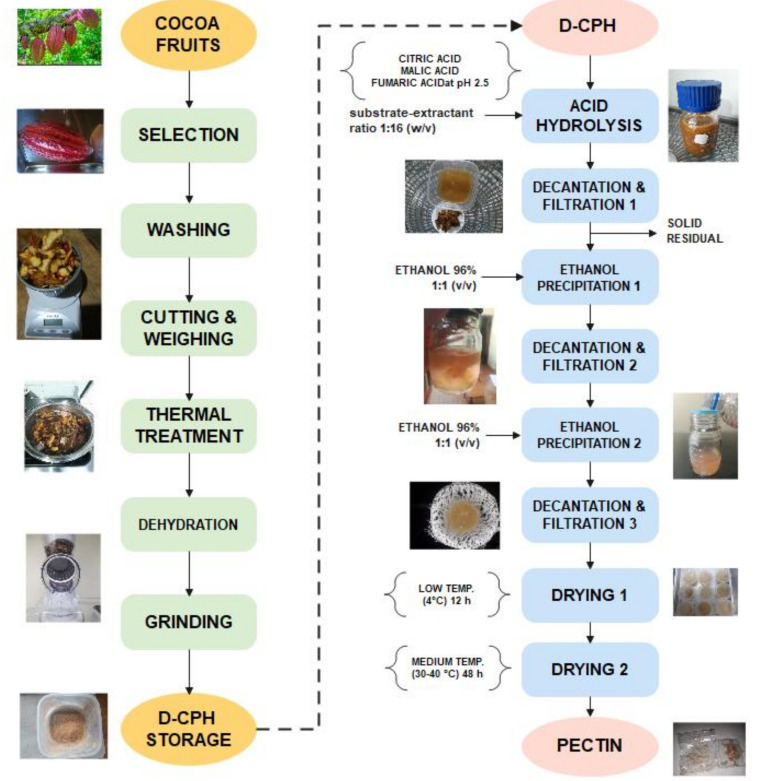
Diagram of the process for obtaining dehydrated CPH (left side) and pectin from it (right side).

Finally, the dehydrated-CPH (d-CPH) residues were crushed in an Oster mill until obtaining a low moisture content. The powder was stored in vacuum-sealed polyethene bags and stored at 4 °C until used in acid hydrolysis experiments (Figure 1, left side).

### 2.4. Pectin Extraction Experiments from Dehydrated CPH

The acid solutions were prepared by diluting about 20 g of each acid with deionised water until an acid solution of pH 2.5 was obtained, according to the procedure described elsewhere [33]. 

Subsequently, 6 g of d-CPH was placed in 100 mL Schoot-flasks, and the acid solution was added in a proportion of 16 mL for each gram of d-CPH (a substrate–extractant ratio of 1:16 (*w*/*v*)), according to the variants of the central composite design (CCD) of experiments to be carried out. 

The temperature of all the experiments was between 70 and 90 °C and was controlled in a recirculating water bath with temperature control (±1 °C). The process times were between 60 and 90 min, according to the CCD variants of experiments (Table 1). 

At the end of each acid extraction time, the flasks were placed in an ice box for about 10 min to rapidly cool the mixture, after which the solid residues were separated from the liquid acid phase. To the latter, a similar volume of separated supernatant was mixed with an equal volume of 96% ethyl alcohol at −15 °C, and the mixture was stirred for a few seconds until it was completely homogeneous. Then, the samples were placed at 4 °C in a conventional refrigerator for about 30 min at rest to facilitate the precipitation of the pectin. 

Finally, the contents of each bottle were carefully decanted and filtered through a white muslin cloth to separate the pectin obtained. The moist pectin was dried in an oven at around 50 °C for 4 h and then weighed on an analytical balance. 

The dry material was stored in the vacuum-sealed polyethene bags at 4 °C until it was used in the characterisation studies carried out later (Figure 1, right side).

The pectin yields obtained (g/kg) by acid hydrolysis were calculated through a modification of the method used by other researchers [33,48]:

(1) $$Yield\;\left(\frac{g}{kg}\right)=\frac{Dry\;pectin\;\left(g\right)}{d-CPH\;\left(kg\right)}$$

### 2.5. Central Composite Design of Experiments Using Response Surface Methodology

To find the combination of temperature, extraction time, and type of acid with which the maximum yield of pectin is obtained, a central composite design (CCD) of experiments using the response surface methodology (RSM) was carried out.

Three factors were altered, two continuous quantitative factors (A: extraction time (min) and B: extraction temperature (°C)), and a nominal qualitative factor (C: type of organic acid). Of the latter, three organic acids were used: C [1] citric acid, C [2] malic acid and C [3] fumaric acid. 

Two blocks of experiments were conducted, which corresponded to two batches of cocoa fruits, all from the same supplier. 

The values of each factor used, and their coded variables, are shown in Table 1.

Design-Expert, release 13.0 (Stat-Ease, Inc., Minneapolis, MN, USA), was the statistical software package employed to manage and analyse the experiments.

The experimental results for CCD using RSM were fit with a second-order polynomial equation using multiple regression techniques.

(2) $$Y=\beta_{0}+\overset{3}{\underset{i=1}{\sum }}\beta_{i}X_{i}+\overset{3}{\underset{i=1}{\sum }}\beta_{ii}X_{i}^{2}+\sum \overset{3}{\underset{i<j=3}{\sum }}\beta_{ij}X_{i}X_{j}+\beta_{123}X_{1}X_{2}X_{3}+\varepsilon =\hat{Y}+\varepsilon$$

where *Y* and $\hat{Y}$ are the response and the “predicted by quadratic model” response (yield of pectin), $\beta_{0}$ is the model intercept coefficient $\beta_{i}$, $\beta_{ii}$ and $\beta_{ij}$ are regression coefficients of the linear, quadratic, and interactive terms, respectively, *X*_*i*_ represents the factors under study (time and temperature of pectin extraction, and organic acid type) and *ε* is the residual error.

### 2.6. Characterisation of CHP-Pectins

#### 2.6.1. FTIR Analysis of CHP-Pectins

The final dry pectin samples, obtained from the acid extractions with the three organic acids in this study, as well as the reference commercial pectin, were analysed by IR spectrometry using an Agilent Cary 630 FTIR (Agilent Technologies Inc., Santa Clara, CA, USA) in a wavenumber range between 400 and 4000 cm^−1^ over 32 scans with a resolution of 4 cm^−1^. In addition, an ATR sampling technique was used on a single rebound diamond crystal.

#### 2.6.2. Determination of Equivalent Weight, Methoxyl Content (MeO), Anhydrouronic Acid Content (AUA) and Degree of Esterification (DE) of CHP-Pectins

Equivalent weight was determined according to the methodology described elsewhere [49,50]. First, 500 mg of dry pectin sample from dehydrated CPH was moistened with 2 mL of ethanol and dissolved in 100 mL of deionised CO_2_-free sterile water. Then, 1 g NaCl and 6 drops of phenol-red indicator were added and mixed vigorously until all the pectin dissolved. After that, the mix was carefully titrated with 0.1 N NaOH until the colour turned pink (pH 7.5).

Equivalent weight was calculated as:

(3) $$Equiv.\;weight\;\left(g/mol\right)=\frac{mass\;sample\;\left(g\right)}{V\left(ml\right)\;alkali\times Conc.\left(N\right)\;alkali}\;$$

Methoxyl (MeO) content was determined according to the methodology described elsewhere [49,50]. Briefly, 25 mL of 0.25 N was added to the neutralised solution described above to determine the equivalent weight. New alkali solutions were mixed well and allowed to stand for half an hour in a stoppered flask. Then, 25 mL of 0.25 N HCl was added and titrated again, as it was made before.

Methoxyl (MeO) content (% wt.) was calculated as:

(4) $$MeO\;\left(\%\right)=\frac{V\left(ml\right)\;alkali\times Conc.\left(N\right)\;alkali\times 31}{mass\;sample\;\left(g\right)\times 1000}\times 100$$

where "31" is the molecular weight of the MeO group.

Anhydrouronic acid (AUA) content (% wt.) in pectin samples was obtained according to the following formula:

(5) $$AUA\;\left(\%\right)=\frac{176\times 0.1\cdot \left(V_{z}+V_{y}\right)}{m\times 1000}\times 100$$

We used the titration volumes of alkali obtained above to determine the equivalent weight ($V_{z}$, mL), methoxyl content ($V_{y}$, mL) and mass of samples (m, g).

Degree of esterification (*DE*) in pectin samples was calculated according to the expression reported by others:

(6) $$DE\left(\%\right)=\frac{176\times MeO\left(\%\right)}{31\times AUA\left(\%\right)}\times 100$$

Degree of esterification (*DE*, %) can also be calculated by determining the peak areas in FTIR spectra of pectin, corresponding to the free carboxyl groups (~1630 cm^−1^) and esterified groups (~1740 cm^−1^), according to the equations [51,52]:

(7) $$R=\frac{A_{1740}}{A_{1740}+A_{1630}}\times 100$$

(8) DE(%)=124.7·R+2.2013

## 3. Results

### 3.1. CCD Experiments and the Model Analysis

CCD experiments to find the best extraction conditions (extraction time and temperature) for each of the three organic acids with GRAS status (citric, malic or fumaric acids) were carried out in two blocks according to the fruits processed to obtain the dehydrated CPH material (d-CPH) that was used as the starting raw material to derive pectin from d-CPH (Table 2).

With the data shown above (Table 2), the following model was obtained, in terms of the codified factors, of the pectin yield:

(9) $$\hat{Y}\left(g/kg\right)=3.81+1.01\cdot A+1.34\cdot C\left[1\right]+0.2857C\left[2\right]\;\;\;\;\left(R^{2}=0.6\right).$$

In terms of actual factors, the equations that best represented the experimental data were:

Citric acid:

(10) $${\hat{Y}}_{CA}\left(g/kg\right)=-1.98440+0.095221\cdot Time\left(min\right)\;\;\;\;$$

Malic acid:

(11) $${\hat{Y}}_{MA}\left(g/kg\right)=-3.04154+0.095221\cdot Time\left(min\right)$$

Fumaric acid:

(12) $${\hat{Y}}_{FA}\left(g/kg\right)=-4.95583+0.095221\cdot Time\left(min\right)\;$$

The analysis of variance (ANOVA) for the yield model showed the significance of each term in the model (Table 3).

**Figure 2:**
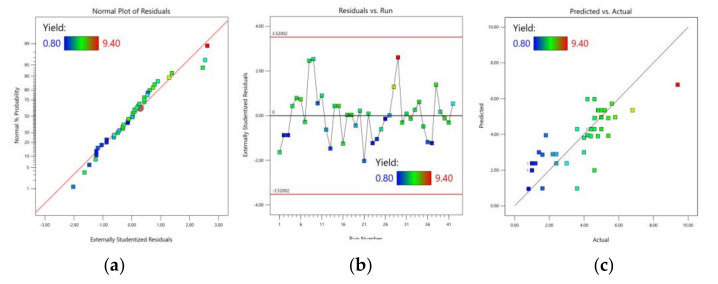
Analysis of the model of the responses for the yield of pectin extraction. (**a**) Normality plot of the residuals for the yield model shown in Equation (7); (**b**) Student’s t external distribution of the residuals; and (**c**) correspondence between the actual values of the yield responses and the values obtained with the model shown in Equation (7).

Additionally, normality (Figure 2a) and the residuals’ distribution (Figure 2b), and correspondence between the values obtained by the model with the actual values (Figure 2c), were checked.

All the analyses showed the usefulness of the pectin yield model and suggested that the yield of the pectin model could be used to find the optimal values.

### 3.2. Optimisation of Pectin Yield Model

We optimised the yield of the pectin model with the maximum levels of importance (5 (+++++)), in which factors A: time and C: organic acid were in range, and a non-significant (*p* > 0.05) temperature factor (B: temp.) was equal to 80 °C (Table 4).

After performing the optimal procedure, three maximum values for the pectin yield were obtained for each organic acid used in this study (Table 5).

**Figure 3:**
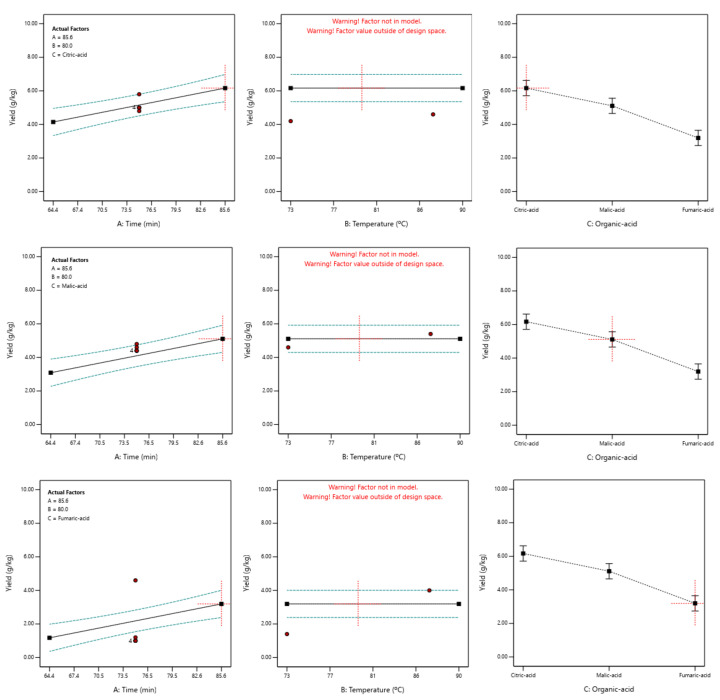
Values of the pectin yield model in relation to factors A: time, B: temp. and C: organic acid for the three organic acids used in this study. Upper: citric acid; middle: malic acid; bottom: fumaric acid.

These optimal values were represented graphically for each of the factors under study (Figure 3).

Moreover, the yield models for each of the three organic acids used in the present study suggested that only the extraction time and the acid type significantly influence (*p* < 0.05) the extraction yield.

### 3.3. Confirmation Experiments for the Pectin Yield Model

Six complementary, confirmatory experiments were carried out in unison and under the conditions in Table 5 (for an extraction time and temperature of around 86 min and 80 °C, respectively) to verify the validity of the obtained model. The pectin yields for each of the organic acids were within the ranges suggested by the models’ Equations (10)–(12) (Table 6).

The complementary validation experiments corroborate that there are significant differences in the acid extraction yield between the three organic acids used in this study, although the real values obtained are somewhat lower than those of the models for citric and malic acid, and somewhat higher than the ones predicted for fumaric acid (Table 6).

### 3.4. Characterisation of the Pectin from d-CPH Using Three Organic Acids

The pectin samples obtained from the confirmatory experiments were used to carry out characterisation studies.

**Figure 4:**
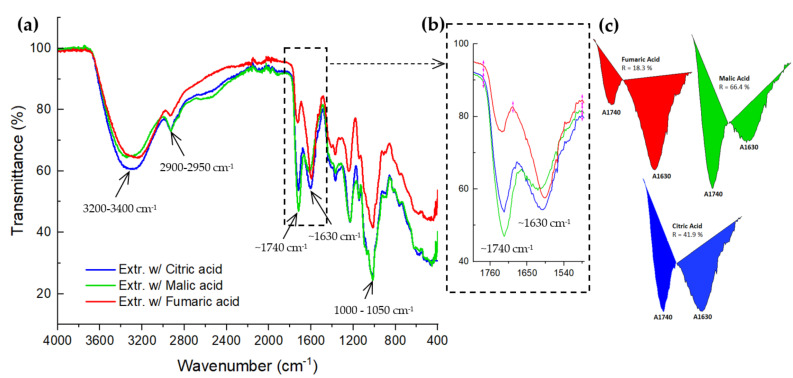
FTIR spectra of the pectin obtained from d-CPH and (**a**) extracted with the different organic acids employed in this study. (**b**) The peaks and their areas are analysed in the zones close to ~1740 and ~1630 cm^−1^, which correspond to the esterified and free carboxyl groups, respectively. (**c**) Areas at ~1740 cm^−1^ and ~1630 cm^−1^ for each FTIR spectrum and the calculation of the R-value (Equation (7)).

FTIR spectra of the pectin samples obtained from d-CPH and extracted with three GRAS-type organic acids demonstrated chemical similarity. They were also similar to those of the FTIR spectrum of the commercial pectin used as a reference and shown elsewhere [52] (Figure 4).

**Figure 5:**
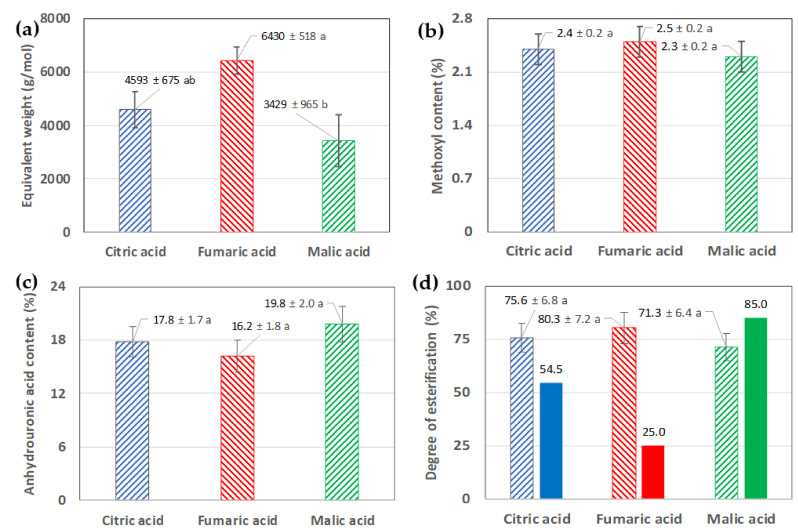
Characterisation of the pectin obtained from d-CPH and extracted with different organic acids employed in this study (citric, malic and fumaric acid). (**a**) Equivalent weight (g/mol); (**b**) methoxyl (MeO) content (%); (**c**) anhydrouronic acid (AUA) content (%); (**d**) degree of esterification (DE, %). The bars filled with a pattern represent the DE (%) values determined according to the titration method (Equation (6)), while the full-coloured bars are the results calculated from the FTIR spectra and the calculation of the areas (A_1740_ and A_1630_, Figure 4b) and Equations (7) and (8). All values: mean ± standard deviation (*n* = 3). In each graph, equal letters denote non-statistically significant differences (*p* < 0.05).

Additionally, the equivalent weight, the MeO and AUA contents (%wt.) and the degree of esterification (DE, %) of the pectin samples obtained from d-CPH by extraction with the three organic acids used in this work were determined (Figure 5).

## 4. Discussion

The process of preparation of the CPH and its dehydration could be extended the storage time of this raw material. In this way, the raw material could be available for longer and not only during the harvest periods of the cocoa fruit (Figure 1, left side).

Decantation filtration characterised the extraction process with different GRAS-status organic acids for separation. It was used in the initial solid–liquid separation processes after the extraction time and in the two processes of ethanolic precipitation for the purification of pectin (Figure 1, right side). However, the decantation-filtration process seems less efficient than other methods, such as press filtration or centrifugation.

For the above reasons, the yields obtained in the present study were lower than those obtained by similar studies. For example, pectin yields of 5.55–7.70% [9] and 6.10–9.20% [53] dry wt., and 18.12% [40], 11.52% [54] and 9.00% [55] wet fresh wt. have been reported elsewhere for extraction with citric acid, while in the present work, a modest 0.62% wt. was achieved.

The CCD experiment suggested that the extraction yields under the conditions of the experiment complied with the following relationship *Y*_*CA*_ > *Y*_*MA*_ > *Y*_*FA*_, as shown by the relationships established between the yield models, such as:

(13) $${\hat{Y}}_{MA}\left(g/kg\right)={\hat{Y}}_{CA}\left(g/kg\right)-1.06={\hat{Y}}_{FA}\left(g/kg\right)+1.91$$

When carrying out the model validation experiments (*n* = 6), the models’ predictions were confirmed and it was observed that ${\bar{Y}}_{CA}>{\bar{Y}}_{MA}>{\bar{Y}}_{FA}$, although the differences between the yields were slightly different from those shown with the models (Equation (14)).

(14) $${\bar{Y}}_{MA}\left(g/kg\right)={\bar{Y}}_{CA}\left(g/kg\right)-\left(1.03\pm 0.16\right)={\bar{Y}}_{FA}\left(g/kg\right)+\left(1.17\pm 0.31\right)$$

The behaviour observed when assessing the acid extraction yields needs to be clarified. It is possibly related to the presence of three carboxyl groups in citric acid compared to the two for malic acid and fumaric acid, and the low solubility of fumaric acid (4.9 g/L at 20 °C) compared to malic acid (558.0 g/L at 20 °C) and citric acid (592.0 g/L at 20 °C).

The first confers certain advantages to citric acid compared to the other two acids, so its acid hydrolysis action could be more effective. Meanwhile, the second prevents fumaric acid from remaining soluble throughout the extraction process and may thus exert an effective hydrolysing effect on the d-CPH solid material.

The FTIR spectra of the pectin obtained from d-CPH with the organic acids with GRAS status were very similar (Figure 4) and close to those reported for commercial pectin [9,44,52]. In the FTIR spectra, we observed -OH peaks at 3400–3200 cm^−1^. These peaks represent many polyhydroxy compounds present in pectin (Figure 4a). The −CH, −CH_2_, and −CH_3_ stretches of galacturonic acid methyl esters absorb at 2900–2950 cm^−1^ (Figure 4a). Peaks ~1750 cm^−1^ correspond to the C=O stretch observed in the ester and derived from the acetyl (-COCH_3_) group (Figure 4a). Finally, peaks ~1630 cm^−1^ are related to the -OH tensile vibration band, and the bands at 1000–1050 cm^−1^ belong to C–O bending or stretching (Figure 4a).

Their esterification degrees were calculated based on the areas of the peaks ~1740 cm^−1^ and ~1630 cm^−1^ of the pectin’s FTIR spectra (Figure 4 b). Pectin extracted with citric (DE = 54.5%) and malic (DE = 85%) acids had high esterification degrees (DE > 50%). Meanwhile, pectin extracted with fumaric acid (DE = 25%) could be classified as having a low esterification degree. These results differed from the titration method results, where all the pectins had high esterification degrees (DE ≈ 75%), and all of them were statistically similar (*n* = 3, *p* < 0.05) (Figure 5d).

Regarding the values of equivalent weight, methoxyl content and anhydrouronic acid content (Figure 5) of the pectin obtained by acid extraction with organic acids such as citric acid, malic acid and fumaric acid from d-CPH, these values were different from those reported by other authors. For example, the values obtained for the yield, methoxyl content, degree of esterification and the equivalent weight of the pectin obtained from CPH using hydrochloric acid for 60 min at 80 °C were 5.50–7.70%, 3.51–4.86%, 10.76–19.96% and 663.83–1549.22 g/mol, respectively [9]. It is possible that the use of a strong acid, such as HCl, for less time than that used in the present work results in a lower degree of esterification and higher equivalent weight and methoxyl content.

However, further studies must be carried out to determine the causes of such differences, especially concerning the degree of esterification, which is closely related to pectin’s applications as a gelling and thickening agent.

## 5. Conclusions

In the present work, pectin was obtained from d-CPH using acid extraction with three organic acids with GRAS status. The pectin obtained had lower yields than previous reports, which is attributed to the pectin isolation and purification procedures. However, the yields were significantly different, with citric acid being the highest and fumaric acid the lowest. The pectin obtained was very similar in appearance each time, and the FTIR spectra demonstrated its similarity, as well as being notably similar to the commercial pectin spectra published by other authors.

However, the pectin obtained with fumaric acid showed differences compared to those obtained with citric acid and malic acid concerning the equivalent weight and the degree of esterification, potentially related to the lower solubility of this organic acid.

## Figures and Tables

**Table 1:** Factors used (real and coded) in the CCD experiments of the present study.

	Coded Factors					
	−1.414	−1.000	0.000		+1.000	+1.414
A: Time (min)	60	64	75		86	90
B: Temperature (°C)	70	73	80		87	90
C: Organic acid	C [1] citric acid		C [2] malic acid		C [3] fumaric acid	

**Table 2:** CCD experiments for the maximisation of the yield of pectin.

Std.	Block	Run	A: Time (min)	B: Temperature (°C)	C: Organic-Acid (−)	Yield (g/kg)
32	1	1	86	87	Fumaric acid	4.0
31	1	2	64	87	Fumaric acid	3.6
17	1	3	64	87	Malic acid	2.4
33	1	4	75	80	Fumaric acid	1.0
18	1	5	86	87	Malic acid	5.4
4	1	6	86	87	Citric acid	4.6
21	1	7	75	80	Malic acid	4.4
29	1	8	64	**73**	Fumaric acid	1.6
5	1	9	75	80	Citric acid	5.0
1	1	10	64	**73**	Citric acid	4.2
15	1	11	64	**73**	Malic acid	2.2
6	1	12	75	80	Citric acid	5.0
19	1	13	75	80	Malic acid	4.8
16	1	14	86	**73**	Malic acid	4.6
3	1	15	64	87	Citric acid	1.8
35	1	16	75	80	Fumaric acid	1.0
30	1	17	86	**73**	Fumaric acid	1.4
2	1	18	86	**73**	Citric acid	4.2
20	1	19	75	80	Malic acid	4.4
34	1	20	75	80	Fumaric acid	4.6
7	1	21	75	80	Citric acid	5.8
23	2	22	90	80	Malic acid	5.6
40	2	23	75	80	Fumaric acid	1.0
28	2	24	75	80	Malic acid	4.4
9	2	25	90	80	Citric acid	9.4
10	2	26	75	70	Citric acid	6.8
22	2	27	60	80	Malic acid	1.6
12	2	28	75	80	Citric acid	5.0
38	2	29	75	70	Fumaric acid	2.4
24	2	30	75	70	Malic acid	3.6
14	2	31	75	80	Citric acid	4.8
41	2	32	75	80	Fumaric acid	1.2
26	2	33	75	80	Malic acid	4.6
11	2	34	75	90	Citric acid	5.2
37	2	35	90	80	Fumaric acid	4.0
8	2	36	60	80	Citric acid	5.4
39	2	37	75	90	Fumaric acid	3.0
13	2	38	75	80	Citric acid	5.0
25	2	39	75	90	Malic acid	5.0
27	2	40	75	80	Malic acid	4.4
42	2	41	75	80	Fumaric acid	1.0
36	2	42	60	80	Fumaric acid	0.8

**Table 3:** ANOVA for CCD of experiments on the yield of pectin extraction.

Source	Sum of Squares	df	Mean Square	F-Value	*p*-Value	
Block	1.60	1	1.60			
Model	88.00	3	29.33	20.70	<0.0001	*significant*
A-Time	24.48	1	24.48	17.28	0.0002	
C-OA ^1^	63.52	2	31.76	22.41	<0.0001	
Residual	52.43	37	1.42			
Lack of Fit	43.18	25	1.73	2.24	0.0728	*not significant*
Pure Error	9.25	12	0.7711			
Cor. Total	142.03	41				

^1^ OA: organic acid.

**Table 4:** Search criteria for the optimal condition for the yield of pectin extraction.

Factor	Goal	Lower Limit	Upper Limit	Importance
A: Time	In range	64.39	85.61	3
B: Temperature	Equal to 80.0	72.93	90.00	3
C: Organic acid	In range	Citric acid	Fumaric acid	3
Yield	Maximise	0.80	9.40	5

**Table 5:** Optimum values of pectin yield obtained with the constraints shown in Table 4.

Experiment	Time	Temperature	Organic Acid	$\hat{Y}\left(g/kg\right)$
1	85.607	80.00	Citric acid	6.167
2	85.607	80.00	Malic acid	5.110
3	85.607	80.00	Fumaric acid	3.196

**Table 6:** Confirmatory experiments of the validity of the pectin yield model for each of the organic acids used in this study.

Organic Acid	Predicted Mean	Std. Dev.	n	SE Pred.	95% PI Low	Data Mean	95% PI High
Citric acid	6.17	1.19	6	0.63	4.89	5.73	7.44
Malic acid	5.11	1.19	6	0.63	3.83	4.7	6.39
Fumaric acid	3.2	1.19	6	0.63	1.92	3.53	4.47

## Data Availability

Data is contained within the article..
